# Influence of Diagnostic and Treatment Delays on Survival Outcomes in Patients With Locally Advanced Rectal Cancer: A Multi‐Center Retrospective Cohort Study

**DOI:** 10.1002/cam4.71744

**Published:** 2026-03-23

**Authors:** Yutian Zhao, Jiahao Zhu, Benjie Xu, Peipei Shen, Fei Xu, Bo Yang, Shengjun Ji, Leyuan Zhou

**Affiliations:** ^1^ Department of Radiotherapy and Oncology Affiliated Hospital of Jiangnan University Wuxi Jiangsu People's Republic of China; ^2^ Department of Outpatient Chemotherapy Harbin Medical University Cancer Hospital Harbin Heilongjiang People's Republic of China; ^3^ Department of Basic Medicine Wuxi School of Medicine, Jiangnan University Wuxi Jiangsu People's Republic of China; ^4^ Department of Radiotherapy and Oncology Suzhou Municipal Hospital, the Affiliated Suzhou Hospital of Nanjing Medical University, Gusu School, Nanjing Medical University Suzhou Jiangsu People's Republic of China

**Keywords:** diagnostic delay, neoadjuvant therapy, prognosis, rectal cancer, treatment delay

## Abstract

**Objective:**

To investigate the impact of diagnostic and treatment delays on the prognosis of patients with locally advanced rectal cancer (LARC).

**Methods:**

Retrospective analyses were performed in Chinese and American cohorts. The intervals from symptom onset to diagnosis and from diagnosis to initial treatment were defined as diagnostic and treatment delays. A univariate and multivariable Cox regression model was conducted for overall survival (OS), cancer‐specific survival (CSS), and disease‐free survival (DFS).

**Results:**

A total of 450 Chinese and 5810 American patients were included in this study. Multivariable analysis revealed that a prolonged diagnostic delay (> 5 months) was significantly associated with decreased OS (hazard ratio [HR]: 1.50, *p* = 0.041) and DFS (HR: 1.36, *p* = 0.047). Additionally, an extended treatment delay (> 2 months) was closely associated with reduced OS (> 1 and ≤ 2 months vs. ≤ 1 month, HR: 1.06, *p* = 0.386; > 2 months vs. ≤ 1 month, HR: 1.52, *p* < 0.001) and CSS (> 1 and ≤ 2 months vs. ≤ 1 month, HR: 1.03, *p* = 0.693; > 2 months vs. ≤ 1 month, HR: 1.58, *p* = 0.001) in patients with LARC.

**Conclusion:**

Diagnostic intervals exceeding 5 months and treatment delays of more than 2 months are significantly associated with unfavorable survival outcomes in LARC patients. These findings underscore the importance of timely diagnostic pathways and treatment initiation and may serve as clinically meaningful benchmarks for healthcare systems to optimize referral processes, resource allocation, and quality‐of‐care assessment in rectal cancer management.

**Abbreviations**
CEA: carcinoembryonic antigen
CI: confidence interval
CRC: colorectal cancer
CSS: cancer‐specific survival
DFS: disease‐free survival
HR: hazard ratio
ICD‐O‐3: International Classification of Diseases
LARC: locally advanced rectal cancer
LDDG: long diagnostic delay group
LTDG: long treatment delay group
MTDG: middle treatment delay group
NCRT: neoadjuvant chemo‐radiotherapy
OS: overall survival
pCR: pathologic complete response
SDDG: short diagnostic delay group
SEER: Surveillance, Epidemiology, and End Results Program
STDG: short treatment delay group

## 1 Introduction

Colorectal cancer (CRC) ranks as the third most commonly diagnosed malignant neoplasm worldwide [1]. Rectal cancer constitutes approximately 30% of all CRC cases, with a majority of patients being diagnosed in the middle to late stages of the disease [2]. Due to the high rates of local recurrence and metastasis, locally advanced rectal cancer (LARC) requires multimodal treatment approaches. The utilization of neoadjuvant chemo‐radiotherapy (NCRT) followed by total mesorectal excision has been shown to decrease local pelvic recurrence rates to less than 10%. However, distant metastasis rates remain elevated, and the 5‐year overall survival (OS) rate remains unsatisfactory [3]. Early diagnosis, timely initiation of treatment, and the optimization of therapeutic strategies are considered critical determinants of improved prognosis. Recently, delays in the management of CRC have become a significant clinical concern, particularly regarding the interval between symptom onset and initial diagnosis (diagnostic delay), as well as the time between diagnosis and definitive surgery (treatment delay).

Diagnostic and treatment delays are generally considered adverse prognostic factors in cancer. However, several studies have reported counterintuitive findings in colorectal cancer (CRC). For example, Singh et al. found no significant negative clinical effect associated with an increased time to diagnosis [4], a similar result was also observed in an earlier report by Stapley [5]. Furthermore, two additional studies found that shorter diagnostic intervals were associated with higher mortality rates in CRC [6, 7]. Regarding treatment delay, two recent studies demonstrated that a prolonged preoperative treatment delay did not result in poorer survival among CRC patients undergoing direct curative surgical treatment [8, 9]. Similarly, Yu et al. reported that CRC patients who received immediate treatment after diagnosis (< 1 month) experienced the worst prognosis compared to those with longer delays [10]. These unexpected outcomes may be attributed to the combined analysis of colon and rectal cancers, as well as differences in disease stage. Indeed, diagnostic intervals have been shown to be significantly associated with higher mortality in rectal cancer but not in colon cancer [11]. A recent systematic review and another study indicated that disease stage may serve as an important confounding factor when analyzing the association between diagnostic or treatment delays and survival in CRC [12, 13]. Moreover, treatment strategies for CRC vary considerably depending on tumor stage and location, particularly in cases of locally advanced disease.

The contrasting and often conflicting findings reported in previous research highlight the need for a more comprehensive investigation into how diagnostic and treatment delays influence survival outcomes in CRC. Importantly, the American cohort does not include diagnostic delay information and cannot be directly compared with the Chinese cohort. This limitation should be considered when interpreting cross‐country comparisons. Therefore, the present study aims to clarify the impact of diagnostic and treatment delays on the prognosis of patients with LARC undergoing standard neoadjuvant therapy followed by surgery.

## 2 Methods

### 2.1 Patients Selection

A retrospective study was conducted using data from the Harbin Medical University Cancer Hospital, the Affiliated Hospital of Jiangnan University, and the Affiliated Suzhou Hospital of Nanjing Medical University, involving 450 Chinese patients with LARC out of a total of 2173 inpatient cases between January 2010 and October 2019. Patients were selected based on the following criteria: (1) clinical stage II‐III (cT2‐4 and/or cN1‐2) classified with magnetic resonance imaging and/or computed tomography; (2) time of diagnosis from 2010.01 to 2021.01; (3) age 20–85 years; (4) adenocarcinoma or mucinous adenocarcinoma; (5) rectal cancer as the only diagnosed cancer; (6) standard neoadjuvant treatment was conducted. The present study adhered to the Declaration of Helsinki and was approved by the Affiliated Hospital of Jiangnan University, the Affiliated Suzhou Hospital of Nanjing Medical University, and the Harbin Medical University Cancer Hospital.

**FIGURE 1:**
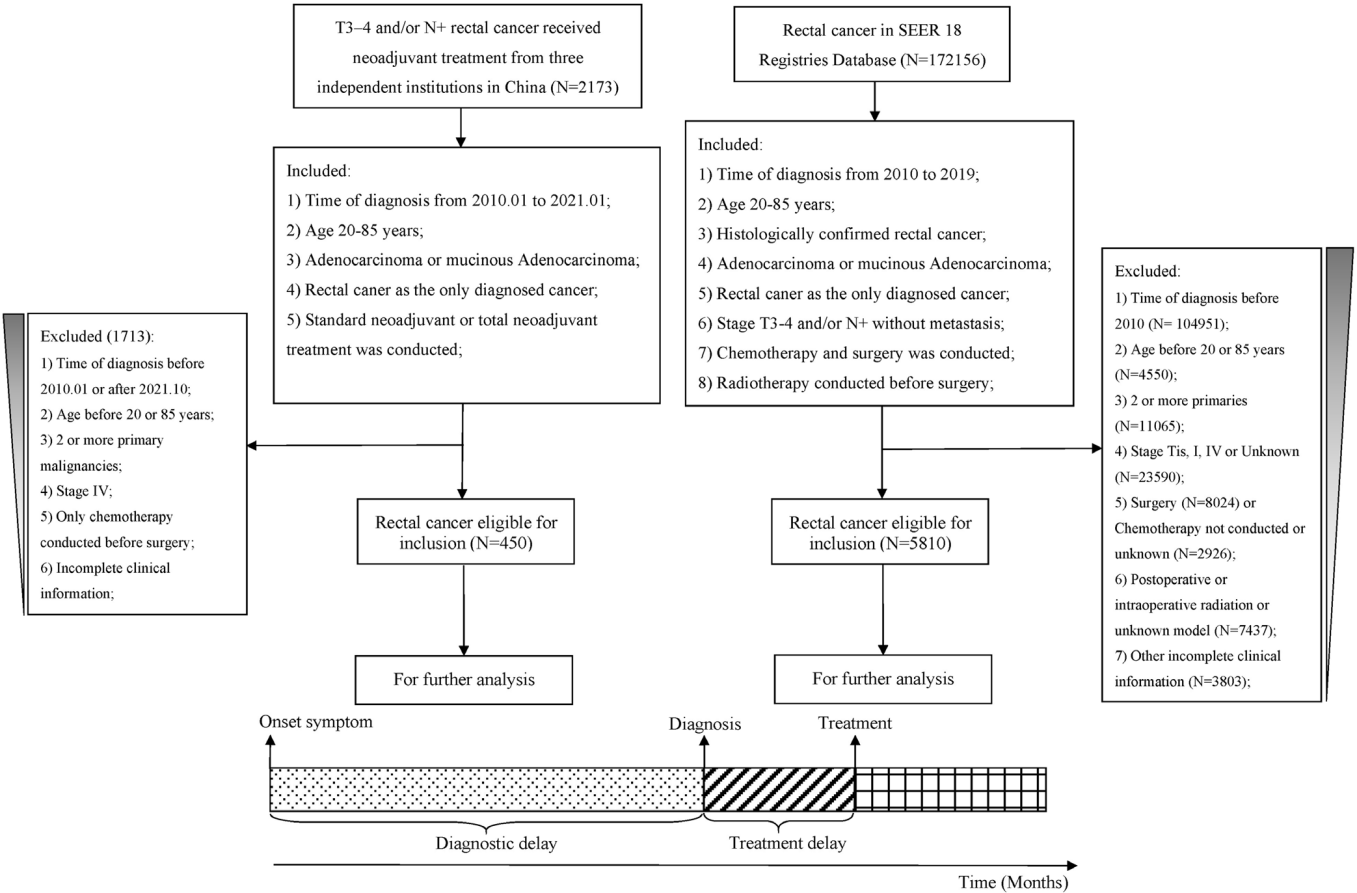
Flow diagram of patient selection and definition of time interval.

We identified 5810 American patients with LARC who received chemoradiotherapy followed by surgery from the 18 population‐based Surveillance, Epidemiology, and End Results Program (SEER) registries between 2010 and 2019. Patients with LARC were identified with topographical and histological codes from the International Classification of Diseases (ICD‐O‐3). C20.9 were the topographical codes, and histological codes were 8140/3 (adenocarcinoma) and 8480/3 (mucinous adenocarcinoma). The inclusion criteria were as follows: (1) time of diagnosis from 2010 to 2019; (2) age 20–85 years; (3) histologically confirmed rectal cancer; (4) adenocarcinoma or mucinous adenocarcinoma; (5) rectal cancer as the only diagnosed cancer; (6) stage T3–4 and/or *N*+ without metastasis; (7) chemotherapy and surgery were performed; (8) radiotherapy was administered before surgery. The detailed selection process is described in Figure 1.

### 2.2 Clinical Indicator Definition

In this study, diagnostic delay was defined as the interval between the date of onset of symptoms (abdominal/rectal pain, blood in stool, weakness/fatigue, weight loss, diarrhea, change in bowel habits, and constipation) and the date of diagnosis (defined as the date of histopathological confirmation through biopsy). Patients with diagnostic delay beyond the threshold were defined as the long diagnostic delay group (LDDG); otherwise, they were defined as the short diagnostic delay group (SDDG). Diagnostic delay information was available only in the Chinese cohort. Because the date of symptom onset was based on patient recall, cases with completely missing onset information were excluded from the diagnostic delay analysis. For patients who reported an approximate timeframe rather than an exact date, the earliest reasonable onset date within the provided range was used to minimize underestimation of the diagnostic interval. Treatment delay was defined as the interval between the date of diagnosis and the initiation of treatment (neoadjuvant chemoradiotherapy). According to the National Accreditation Program for Rectal Cancer guidelines, patients with treatment delay ≤ 1 month were defined as a short treatment delay group (STDG), > 2 months as a long treatment delay group (LTDG), and the rest as a middle treatment delay group (MTDG) [14]. In the American cohort, treatment‐delay times were highly concentrated around 1–2 months, and national guidelines already recommend treatment initiation within 2 months, providing a clinically established and widely accepted benchmark.

### 2.3 Follow‐Up and Outcomes

Postoperative follow‐up visits were scheduled every 6 months for the first 3 years, followed by annual visits thereafter. Regular examinations included physical examinations, laboratory tests, colonoscopy, and imaging. The primary endpoints were OS, cancer‐specific survival (CSS), and disease‐free survival (DFS). Pathologic complete response (pCR) was determined as the secondary endpoint. OS was the interval from the time in months from the date of surgery to death or the last visit. CSS was the interval from the surgery to the occurrence of a LARC‐specific death. DFS was the interval from the surgery to the disease recurrence. pCR refers to the absence of evidence of residual tumor cells in the surgical specimens from both the primary site and resected lymph nodes.

### 2.4 Statistical Analyses

The chi‐square test, or Fisher's exact test, was employed to compare the characteristics among different groups. For continuous variables, the Student's *t*‐test and Wilcoxon's rank‐sum test were performed. Kaplan–Meier curves were drawn, and then the differences were evaluated with the log‐rank test. X‐tile, a graphical tool designed for assessing the optimal cut‐point of a continuous variable, was used to determine the optimal dichotomous cutoffs of diagnostic and treatment delay according to OS [15]. We applied univariate and multivariable Cox regression to assess the relationship between survival outcomes and clinically relevant variables. A two‐sided *p* < 0.05 was considered to be statistically significant. R software version 4.2.0 and X‐tile were used for all statistical analyses.

## 3 Results

### 3.1 Patient Characteristics

A total of 450 Chinese and 5810 American patients were recruited for this study. The median time from symptom onset to diagnosis was 5 months, with an interquartile range (IQR) of 3–6 months in the Chinese cohort. The median time from diagnosis to treatment was 8 days with an IQR of 5–13 days in the Chinese cohort and 1 month with an IQR of 1–2 months in the American cohort. The treatment delay of 21 (4.7%) patients who received initial neoadjuvant chemotherapy or chemoradiotherapy exceeded 1 month, and no cases waited more than 2 months in the Chinese cohort. However, the treatment delay of 2028 (34.9%) patients exceeded 1 month, and 365 (6.8%) cases waited more than 2 months in the American cohort. Chinese patients with LARC experienced shorter treatment delay than American individuals (*p* < 0.001). Over the entire follow‐up period, a total of 104 patients (23.1%) experienced mortality. 162 patients (36.0%) experienced disease recurrence in the Chinese cohort, and 1107 patients (19.1%) experienced mortality in the American database.

Finally, 5 months was defined as the optimal binary threshold of diagnostic delay with X‐tile in the Chinese cohort (Figures S1–S5). Therefore, LARC patients with treatment delay ≤ 5 months were defined as the SDDG and > 5 months as the LDDG. However, no reasonable optimal binary threshold of treatment delay could be obtained in the Chinese cohort. Therefore, Chinese patients were divided into subgroups, as Americans recommend. Additionally, given the lack of information about diagnostic delay in the American database, the Chinese and American cohorts were mainly used to explore the influence of diagnostic and treatment delay on survival outcomes in LARC patients, respectively. No significant differences in clinical characteristics were noted between SDDG and LDDG in the Chinese cohort. Nevertheless, differences in age, year of diagnosis, and tumor differentiation existed among the three treatment delay groups in the American cohort. The main clinical characteristics of the patients are shown in Tables 1 and S1.

**TABLE 1:** Baseline characteristics of chinese and american patients with locally advanced rectal cancer.

Characteristics	Chinese cohort (*n* = 450)			American cohort (*n* = 5810)			
	SDDG (*n* = 289)	LDDG (*n* = 161)	*P*	STDG (*n* = 3782)	MTDG (*n* = 1663)	LTDG (*n* = 365)	*P*
Gender			0.841				0.644
Female	100 (34.6%)	58 (36.0%)		1,416 (37.4%)	602 (36.2%)	132 (36.2%)	
Male	189 (65.4%)	103 (64.0%)		2,366 (62.6%)	1,061 (63.8%)	233 (63.8%)	
Age (Years)			0.789				< 0.001
Median (IQR)	58 (53 to 63)	58 (51 to 65)		57 (50 to 66)	59 (52 to 67)	61 (53 to 67)	
Year of diagnosis			0.515				< 0.001
2010–2013	14 (4.8%)	13 (8.1%)		1,382 (36.5%)	475 (28.6%)	101 (27.7%)	
2014–2016	90 (31.1%)	48 (29.8%)		1,254 (33.2%)	540 (32.5%)	105 (28.8%)	
2017–2019	185 (64.0%)	100 (62.1%)		1,146 (30.3%)	648 (39%)	159 (43.6%)	
Histology			0.904				0.664
Adenocarcinoma	275 (95.2%)	152 (94.4%)		3,657 (96.7%)	1,600 (96.2%)	352 (96.4%)	
Mucinous adenocarcinoma	14 (4.8%)	9 (5.6%)		125 (3.3%)	63 (3.8%)	13 (3.6%)	
Differentiation			0.986				0.006
Well	30 (10.4%)	16 (9.9%)		295 (7.8%)	147 (8.8%)	29 (7.9%)	
Moderate	228 (78.9%)	128 (79.5%)		3,061 (80.9%)	1,376 (82.7%)	309 (84.7%)	
Poor	31 (10.7%)	17 (10.6%)		426 (11.3%)	140 (8.4%)	27 (7.4%)	
Pretreatment CEA			0.336				0.405
Normal	161 (55.7%)	98 (60.9%)		2117 (56%)	920 (55.3%)	216 (59.2%)	
Elevated	128 (44.3%)	63 (39.1%)		1665 (44%)	743 (44.7%)	149 (40.8%)	
cT stage			0.462				0.205
cT2	12 (4.2%)	7 (4.3%)		221 (5.8%)	97 (5.9%)	26 (7.2%)	
cT3	201 (69.6%)	103 (64%)		3,115 (82.4%)	1,384 (83.2%)	283 (77.5%)	
cT4	76 (26.3%)	51 (31.7%)		446 (11.8%)	182 (10.9%)	56 (15.3%)	
cN stage			0.203				0.051
cN0	98 (33.9%)	49 (30.4%)		1,243 (32.9%)	570 (34.3%)	144 (39.5%)	
cN1	132 (45.7%)	87 (54%)		1,940 (51.3%)	862 (51.8%)	172 (47.1%)	
cN2	59 (20.4%)	25 (15.5%)		599 (15.8%)	231 (13.9%)	49 (13.4%)	
cTNM			0.464				0.094
IIA	75 (26%)	33 (20.5%)		1,104 (29.2%)	519 (31.2%)	125 (34.2%)	
IIB	17 (5.9%)	14 (8.7%)		107 (2.8%)	39 (2.3%)	12 (3.3%)	
IIC	6 (2.1%)	2 (1.2%)		32 (0.8%)	12 (0.7%)	7 (1.9%)	
IIIA	12 (4.2%)	7 (4.3%)		186 (4.9%)	86 (5.2%)	21 (5.8%)	
IIIB	113 (39.1%)	74 (46%)		1,850 (48.9%)	807 (48.5%)	165 (45.2%)	
IIIC	66 (22.8%)	31 (19.3%)		503 (13.3%)	200 (12%)	35 (9.6%)	
pPNI			0.557				0.911
Negative	268 (92.7%)	146 (90.7%)		3,320 (87.8%)	1,458 (87.7%)	323 (88.5%)	
Positive	21 (7.3%)	15 (9.3%)		462 (12.2%)	205 (12.3%)	42 (11.5%)	
pTDs			0.881				0.239
Negative	223 (77.2%)	126 (78.3%)		3,314 (87.6%)	1,442 (86.7%)	328 (89.9%)	
Positive	66 (22.8%)	35 (21.7%)		468 (12.4%)	221 (13.3%)	37 (10.1%)	

Abbreviations: CEA: Carcinoembryonic antigen; cN: Clinical lymph node; cT: Clinical tumor; cTNM: Clinical tumor, lymph node, and metastasis system stage; lymph node, and metastasis; IQR: Interquartile range; LDDG: Long diagnostic delay group; LTDG: Long treatment delay group; MTDG: Middle treatment delay group; PNI: Perineural invasion; SDDG: Short diagnostic delay group; STDG: Short treatment delay group; TDs: Tumor deposits.

### 3.2 Prognosis

**FIGURE 2:**
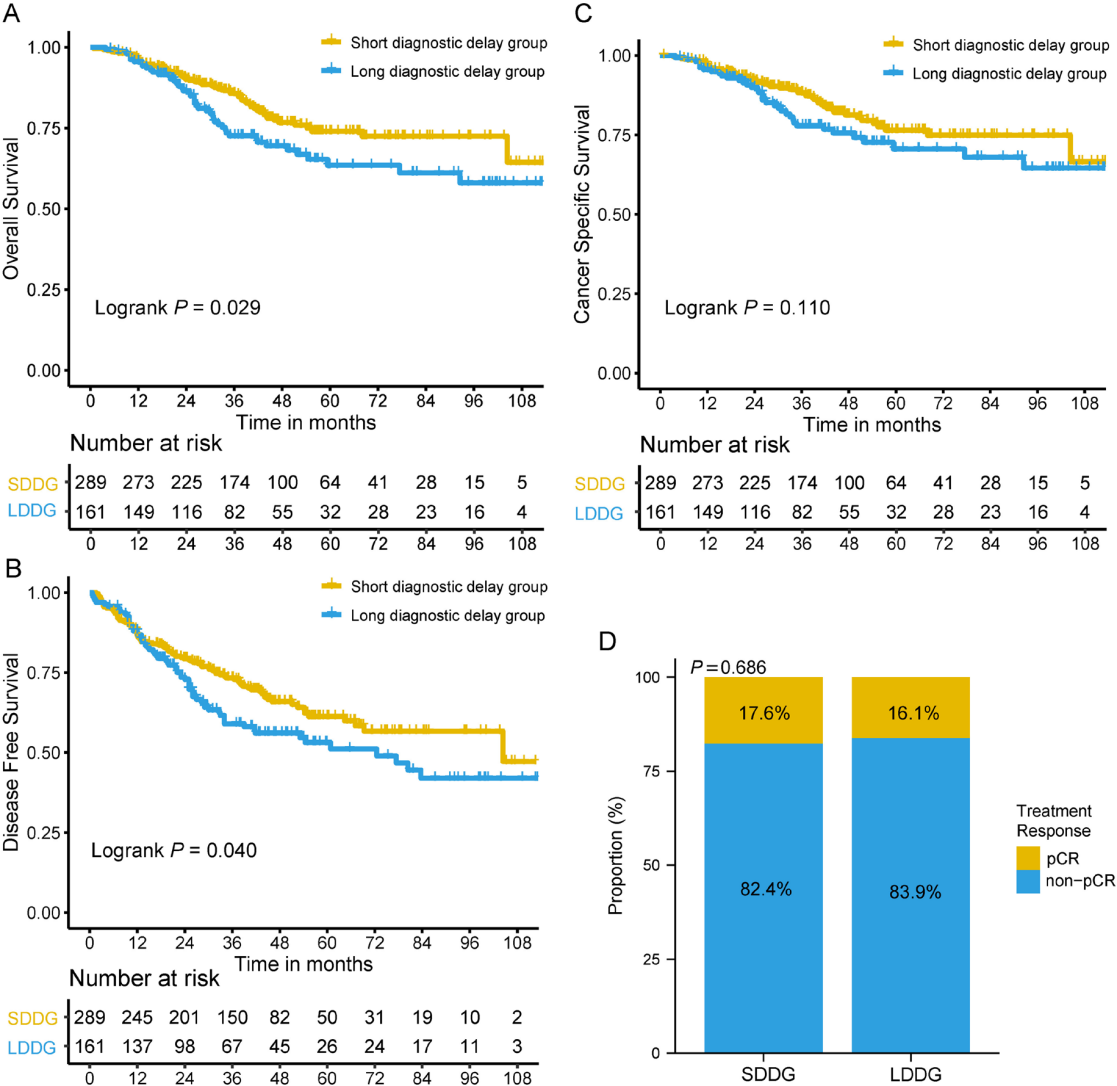
Differences in survival and treatment response between the short diagnostic delay group (SDDG) and the long diagnostic delay group (LDDG) in Chinese locally advanced rectal cancer patients. (A) Overall survival. (B) Disease‐free survival. (C) Cancer‐specific survival. (D) Pathologic complete response (pCR).

**FIGURE 3:**
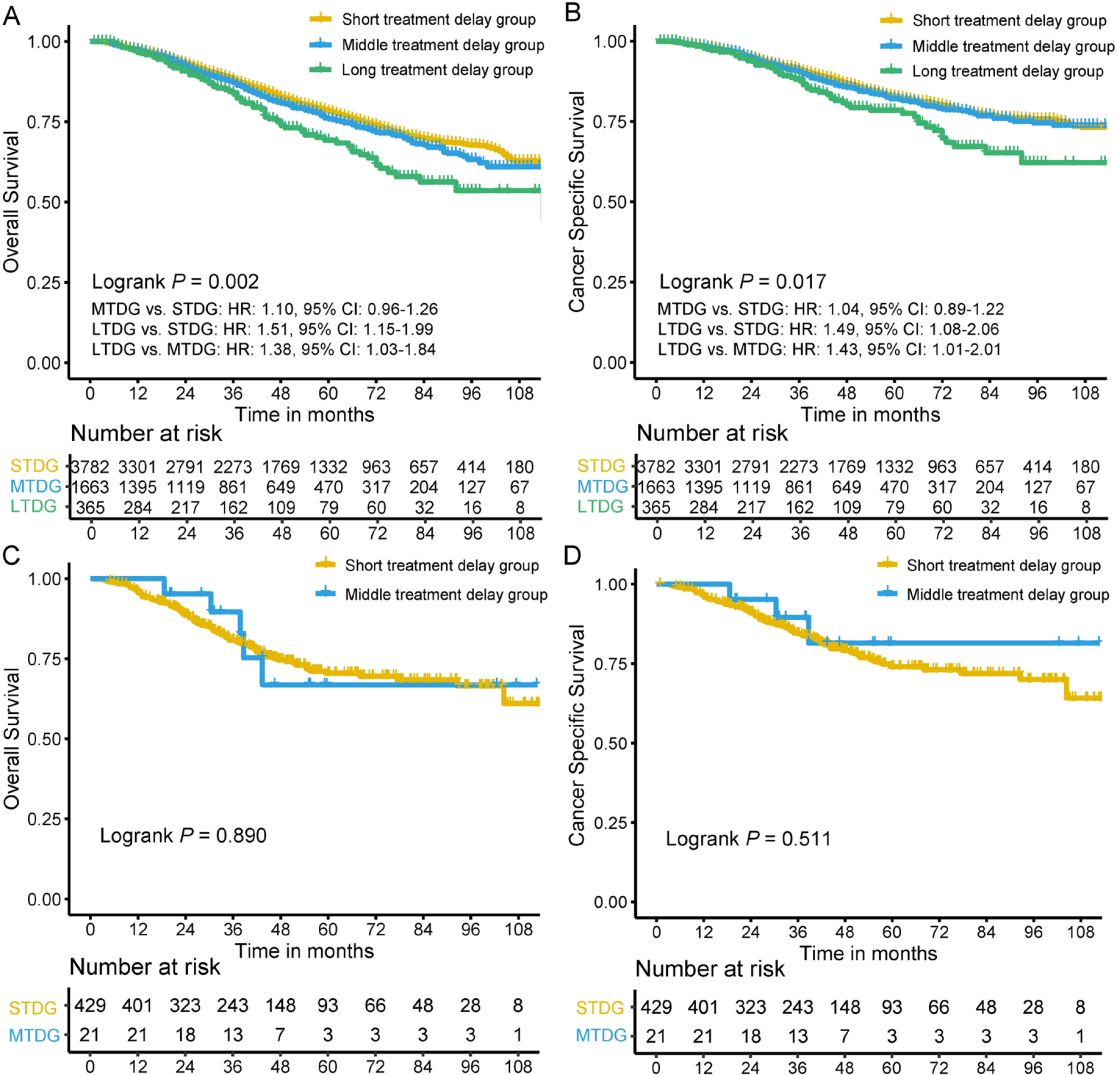
Differences in survival among the short treatment delay group (STDG), middle treatment delay group (MTDG), and long treatment delay group (LTDG) in American and Chinese locally advanced rectal cancer patients. (A, B) Overall survival and cancer‐specific survival in the American cohort. (C, D) Overall survival and cancer‐specific survival in the Chinese cohort.

There were significant differences in the Chinese cohort between SDDG and LDDG in OS (Figure 2A, *p* = 0.029) and DFS (Figure 2B, *p* = 0.040), while not in CSS (Figure 2C, *p* = 0.110) or pCR (Figure 2D, *p* = 0.686). Significant differences in the American database were also observed among STDG, MTDG, and LTDG in OS (Figure 3A, *p* = 0.002) and CSS (Figure 3B, *p* = 0.017). Further comparisons of the three groups showed that the main differences between OS and CSS were concentrated between STDG, MTDG, and LTDG, but not between STDG and MTDG. A similar relationship between STDG and MTDG in OS (Figure 3C, *p* = 0.890) and CSS (Figure 3D, *p* = 0.511) was also found in the Chinese cohort. No significant difference existed in pCR between STDG and MTDG (Figure S6, *p* = 0.890).

Multivariate analysis showed that age (hazard ratio [HR]: 1.33, 95% confidence interval [CI], 1.08–1.44, *p* = 0.026) and diagnostic delay (HR: 1.50, 95% CI, 1.02–2.21, *p* = 0.041) served as the independent prognostic factors for OS, tumor deposits (HR: 1.73, 95% CI, 1.09–2.73, *p* = 0.019) as the only independent prognostic factor for CSS, and tumor deposits (HR: 1.55, 95% CI, 1.11–2.18, *p* = 0.011), N stage (HR: 1.04, 95% CI, 1.00–1.32, *p* = 0.049), and diagnostic delay (HR: 1.36, 95% CI, 1.01–1.85, *p* = 0.047) for DFS (Table 2). In the American database, treatment delay was the independent prognostic factor for OS (MTDG vs. STDG, HR: 1.06, 95% CI, 0.92–1.21, *p* = 0.386; LTDG vs. STDG, HR: 1.52, 95% CI, 1.20–1.92, *p* < 0.001) and CSS (MTDG vs. STDG, HR: 1.03, 95% CI, 0.87–1.21, *p* = 0.693; LTDG vs. STDG, HR: 1.58, 95% CI, 1.20–2.09, *p* = 0.001). Besides treatment delay, gender, age, year of diagnosis, histology, pretreatment carcinoembryonic antigen (CEA), perineural invasion, tumor deposits, T stage, and N stage significantly impacted the OS and CSS (*p* < 0.05). Tumor differentiation also influenced CSS (*p* < 0.05) (Table 3).

**TABLE 2:** Univariate and multivariate cox regression analysis for overall survival (OS), cancer‐specific survival (CSS), and disease‐free survival (DFS) in the chinese cohort.

Characteristics	UVA (OS)		MVA (OS)		UVA (CSS)		MVA (CSS)		UVA (DFS)		MVA (DFS)	
	HR 95% CI	*P*	HR 95% CI	*P*	HR 95% CI	*P*	HR 95% CI	*P*	HR 95% CI	*P*	HR 95% CI	*P*
Gender												
Female	Ref.				Ref.				Ref.			
Male	1.08 (0.73–1.62)	0.694			1.20 (0.77–1.86)	0.427			0.99 (0.71–1.37)	0.951		
Age (Years)	1.27 (1.14–1.56)	0.016	1.33 (1.08–1.44)	0.026	1.01 (0.99–1.04)	0.268			1.01 (0.99–1.02)	0.542		
Year of diagnosis												
2010–2013	Ref.				Ref.				Ref.			
2014–2016	1.16 (0.54–2.52)	0.701			1.09 (0.47–2.52)	0.833			0.95 (0.50–1.78)	0.861		
2017–2019	0.82 (0.38–1.80)	0.627			0.81 (0.35–1.88)	0.619			0.91 (0.48–1.70)	0.757		
Histology												
Adenocarcinoma	Ref.				Ref.				Ref.			
Mucinous adenocarcinoma	0.48 (0.15–1.52)	0.211			0.57 (0.18–1.81)	0.339			0.63 (0.28–1.44)	0.275		
Differentiation												
Well	Ref.				Ref.				Ref.			
Moderate	1.41 (0.68–2.91)	0.357			1.34 (0.62–2.91)	0.462			1.05 (0.63–1.77)	0.845		
Poor	2.15 (0.89–5.20)	0.089			1.73 (0.64–4.66)	0.278			1.18 (0.59–2.33)	0.641		
Pretreatment CEA												
Normal	Ref.				Ref.				Ref.			
Elevated	1.08 (0.73–1.59)	0.698			1.28 (0.84–1.96)	0.258			1.28 (0.94–1.74)	0.118		
Perineural invasion												
Negative	Ref.				Ref.				Ref.			
Positive	1.49 (0.78–2.87)	0.228			1.26 (0.58–2.73)	0.564			1.40 (0.83–2.35)	0.203		
Tumor deposits												
Negative	Ref.		Ref.		Ref.		Ref.		Ref.		Ref.	
Positive	1.57 (1.03–2.39)	0.036	1.48 (0.97–2.25)	0.071	1.73 (1.09–2.73)	0.019	1.73 (1.09–2.73)	0.019	1.61 (1.15–2.26)	0.006	1.55 (1.11–2.18)	0.011
T stage												
T1–T2	Ref.				Ref.				Ref.			
T3–T4	1.59 (0.50–5.02)	0.428			1.92 (0.47–7.82)	0.362			1.88 (0.69–5.06)	0.215		
N stage												
Negative	Ref.				Ref.				Ref.		Ref.	
Positive	1.13 (0.75–1.70)	0.553			1.40 (0.88–2.23)	0.161			1.22 (1.09–1.52)	0.036	1.04 (1.00–1.32)	0.049
Diagnostic delay (Months)												
≤ 5	Ref.		Ref.		Ref.				Ref.		Ref.	
> 5	1.53 (1.04–2.26)	0.031	1.50 (1.02–2.21)	0.041	1.41 (0.92–2.17)	0.116			1.39 (1.01–1.90)	0.041	1.36 (1.01–1.85)	0.047
Treatment delay (Months)												
≤ 1	Ref.				Ref.				Ref.			
> 1	0.94 (0.38–2.31)	0.891			0.68 (0.21–2.15)	0.511			0.69 (0.31–1.56)	0.376		

Abbreviations: CEA: Carcinoembryonic antigen; CI: Confidence interval; CSS: Cancer‐specific survival; DFS: Disease‐free survival; HR: Hazard ratio; N: Lymph node; MVA: Multivariate analysis; OS: Overall survival; Ref: Reference; T: Tumor; TNM: Tumor, lymph node, and metastasis system stage; UVA: Univariate analysis.

**TABLE 3:** Univariate and multivariate Cox regression analysis for overall survival (OS) and cancer‐specific survival (CSS) in the American cohort.

Characteristics	UVA (OS)		MVA (OS)		UVA (CSS)		MVA (CSS)	
	HR 95% CI	*P*	HR 95% CI	*P*	HR 95% CI	*P*	HR 95% CI	*P*
Gender								
Female	Ref.		Ref.		Ref.		Ref.	
Male	1.28 (1.12–1.45)	< 0.001	1.26 (1.11–1.43)	< 0.001	1.20 (1.03–1.39)	0.015	1.19 (1.03–1.38)	0.019
Age (Years)	1.02 (1.02–1.03)	< 0.001	1.03 (1.02–1.03)	< 0.001	1.01 (1.01–1.02)	< 0.001	1.01 (1.01–1.02)	< 0.001
Year of diagnosis								
2010–2013	Ref.		Ref.		Ref.		Ref.	
2014–2016	0.84 (0.73–0.97)	0.017	0.86 (0.75–0.99)	0.037	0.805 (0.68–0.94)	0.009	0.80 (0.68–0.94)	0.009
2017–2019	0.81 (0.64–1.03)	0.091	0.79 (0.62–1.01)	0.058	0.880 (0.66–1.16)	0.364	0.83 (0.63–1.10)	0.207
Histology								
Adenocarcinoma	Ref.				Ref.		Ref.	
Mucinous adenocarcinoma	1.58 (1.23–2.02)	< 0.001	1.39 (1.09–1.79)	0.009	1.56 (1.17–2.10)	0.002	1.35 (1.01–1.82)	0.045
Differentiation								
Well	Ref.				Ref.		Ref.	
Moderate	1.04 (0.83–1.32)	0.712	1.02 (0.81–1.29)	0.895	1.12 (0.84–1.49)	0.439	1.06 (0.79–1.41)	0.679
Poor	1.47 (1.12–1.93)	0.006	1.31 (0.99–1.72)	0.057	1.82 (1.31–2.53)	< 0.001	1.51 (1.08–2.09)	0.015
Pretreatment CEA								
Normal	Ref.		Ref.		Ref.		Ref.	
Elevated	1.57 (1.39–1.76)	< 0.001	1.39 (1.23–1.57)	< 0.001	1.67 (1.45–1.92)	< 0.001	1.42 (1.23–1.64)	< 0.001
Perineural invasion								
Negative	Ref.		Ref.		Ref.		Ref.	
Positive	2.33 (2.01–2.70)	< 0.001	1.87 (1.60–2.19)	< 0.001	2.89 (2.45–3.40)	< 0.001	2.10 (1.76–2.50)	< 0.001
Tumor deposits								
Negative	Ref.		Ref.		Ref.		Ref.	
Positive	2.10 (1.82–2.44)	< 0.001	1.65 (1.40–1.93)	< 0.001	2.55 (2.17–3.01)	< 0.001	1.76 (1.47–2.10)	< 0.001
T stage								
T1–T2	Ref.		Ref.		Ref.		Ref.	
T3–T4	2.14 (1.52–3.01)	< 0.001	1.93 (1.36–2.73)	< 0.001	2.26 (1.49–3.43)	< 0.001	2.13 (1.39–3.24)	< 0.001
N stage								
Negative	Ref.		Ref.		Ref.		Ref.	
Positive	1.27 (1.12–1.44)	< 0.001	1.28 (1.12–1.46)	< 0.001	1.69 (1.44–1.98)	< 0.001	1.59 (1.34–1.88)	< 0.001
Treatment delay (Months)								
≤ 1	Ref.		Ref.		Ref.		Ref.	
> 1, ≤ 2	1.10 (0.96–1.26)	0.157	1.06 (0.92–1.21)	0.386	1.04 (0.88–1.22)	0.598	1.03 (0.87–1.21)	0.693
> 2	1.51 (1.20–1.91)	< 0.001	1.52 (1.20–1.92)	< 0.001	1.49 (1.13–1.96)	0.004	1.58 (1.20–2.09)	0.001

Abbreviations: CEA: Carcinoembryonic antigen; CI: Confidence interval; CSS: Cancer‐specific survival; HR: Hazard ratio; MVA: Multivariate analysis; N: Lymph node; OS: Overall survival; Ref: Reference; T: Tumor; TNM: Tumor, lymph node, and metastasis system stage; UVA: Univariate analysis.

### 3.3 Delay Intervals in Younger and Older Patients

Given that age served as an independent factor for OS in both the Chinese and American cohorts, differences in diagnostic and treatment delay in younger (≤ 50 years) and older (> 50 years) LARC patients were further analyzed. There was no significant difference in diagnostic and treatment delay between younger and older groups in the Chinese cohort. However, patients who were more than 50 years old experienced a longer treatment delay, with a mean difference of 0.08 months (about 2.4 days) in the American population (*p* < 0.001) (Table 4).

**TABLE 4:** Delay Intervals in younger (≤ 50 years) and older (> 50 years) patients with locally advanced rectal cancer.

Population	Variable	Age ≤ 50 years	Age > 50 years	*P*
Chinese cohort	Diagnostic delay (Months)	*N* = 87	*N* = 363	0.122
	Median (IQR)	5 (3–7)	4 (3–6)	
	Mean (SD)	4.89 (2.24)	4.47 (2.26)	
	Range	0–8	0–8	
Chinese cohort	Treatment delay (Days)	*N* = 87	*N* = 363	0.847
	Median (IQR)	8 (5–12)	8 (5–13)	
	Mean (SD)	10.47 (10.65)	10.49 (7.94)	
	Range	2–61	1–59	
American cohort	Treatment delay (Months)	*N* = 1481	*N* = 4329	< 0.001
	Median (IQR)	1 (1–2)	1 (1–2)	
	Mean (SD)	1.33 (0.59)	1.46 (0.69)	
	Range	0–10	0–13	

Abbreviations: IQR: Interquartile range; SD: Standard deviation.

## 4 Discussion

This retrospective study demonstrated that a prolonged diagnostic delay (> 5 months) was associated with worse OS and DFS in LARC patients. Patients with treatment delays exceeding 2 months suffered from poorer OS and CSS. Young patients or those with poor tumor differentiation always had a shorter treatment delay. Moreover, LARC patients in America seemed to have a longer waiting time between diagnosis and initial treatment compared with Chinese patients. No diagnostic or treatment delay difference existed between younger and older LARC patients in the Chinese cohort. Nevertheless, patients who were more than 50 years of age had a longer treatment delay in the American cohort.

Recently, several studies focused on the problems concerning diagnostic and treatment delays in survival in CRC patients due to the COVID‐19 pandemic, which may provide a reference for those patients who could not obtain a timely diagnosis and treatment. Several previous studies were conducted in the Western world to investigate the impact of diagnostic delay on CRC stage and prognosis. A study from the Netherlands showed a median time of 4.6 months between the onset of symptoms and initial treatment [16]. A similar median time of 4.4 months was observed in another study conducted with the Spanish cohort [17]. However, these studies found that longer diagnostic delays had no significant relationship with worse survival or a more advanced CRC stage. On the contrary, an inverse association between diagnostic delay and CRC stage was observed [12, 18]. Additionally, patients with short diagnostic intervals were even found to have a significantly higher mortality rate for rectal cancers [11]. They attributed the so‐called “waiting time paradox” to differences in disease stage at symptom onset, tumor location, and tumor aggressiveness. Our study recruited only LARC patients who received the standard neoadjuvant treatment model, which might reduce the potential confounding factors. Although a mean time of 5 months of diagnostic delay in the Chinese cohort seemed to be close to the data in Western countries, there were wide differences in practice due to variations in healthcare insurance policies, healthcare systems, and medical ecosystems across developed and developing countries. In particular, the shorter treatment delay observed in Chinese patients is likely driven, at least in part, by system‐level characteristics, such as patterns of insurance coverage, centralized delivery of neoadjuvant therapy in high‐volume tertiary centers, and streamlined referral pathways, rather than uniformly greater clinical urgency. Previous studies have also suggested that healthcare organizations, reimbursement models, and resource allocation strongly influence waiting times for colorectal cancer care, sometimes independently of tumor biology or symptom burden [16, 17, 18]. Therefore, the differences in treatment delay between China and the United States in our study should primarily be interpreted as reflections of healthcare system performance, and caution is required when extrapolating these findings to countries with different health system structures. In this study, a prolonged diagnostic delay (> 5 months) in LARC was associated with poor OS and DFS, while having no association with CSS, pCR, or tumor‐related risk factors.

The observed differences in treatment delay intervals between the Chinese and U.S. cohorts should be interpreted primarily as reflections of healthcare system–level and organizational factors rather than intrinsic differences in tumor biology. All patients included in this study had locally advanced rectal cancer and received standardized neoadjuvant chemoradiotherapy followed by surgery, which minimizes biological heterogeneity related to disease stage and treatment modality. The shorter treatment delay observed in Chinese patients is likely influenced by systemic characteristics, such as centralized care delivery in high‐volume tertiary centers, streamlined referral pathways, and differences in insurance coverage and reimbursement policies. In contrast, longer treatment delay in the U.S. cohort may reflect variations in healthcare access, referral coordination, and administrative processes. Therefore, caution is warranted when interpreting cross‐country differences, and these findings should primarily be viewed as indicators of healthcare system performance rather than tumor aggressiveness.

The identified time thresholds may have direct implications for clinical practice and healthcare quality improvement. A diagnostic interval exceeding 5 months may serve as a practical warning signal in routine outpatient settings, prompting earlier referral for colonoscopic evaluation in patients with persistent rectal symptoms. Similarly, a treatment delay beyond 2 months after diagnosis may represent a critical window during which intensified multidisciplinary coordination is warranted to avoid adverse survival outcomes. These thresholds could be integrated into institutional referral pathways or quality‐of‐care benchmarks, enabling hospitals to monitor timeliness of care delivery, identify system‐level delays, and optimize resource allocation. Framing diagnostic and treatment delay as modifiable care‐process indicators rather than fixed patient‐related factors may facilitate earlier intervention, improve patient triage, and ultimately enhance outcomes in locally advanced rectal cancer.

The absence of a significant association between diagnostic or treatment delays and pCR warrants further consideration. Achieving pCR after neoadjuvant chemoradiotherapy is strongly influenced by intrinsic tumor biology, including molecular subtype, tumor microenvironmental features, radiosensitivity, and immune infiltration profiles, many of which are not directly affected by the timing of diagnosis or treatment initiation. Therefore, even substantial variation in clinical delay may not meaningfully alter the probability of achieving pCR, particularly if the delay intervals fall within ranges that do not significantly change tumor biology. Additionally, the relatively small number of pCR events in the Chinese cohort may have reduced statistical power to detect modest associations. Together, these factors suggest that the lack of association observed in our study may be driven by both biological determinants of treatment response and limitations related to sample size.

In terms of treatment delay, institutions in Britain and America recommended initiating treatment for CRC within 2 months of the patient's primary clinical evaluation [19, 20]. From the results of our study with the American database, this suggestion was meaningful in practice. We found that LTDG had lower OS and CSS when compared with STDG and MTDG in LARC patients in the American database. Nevertheless, another study designed similarly to ours did not come to the same conclusion. Edwards et al. investigated whether the therapy within 60 days of diagnosis could influence the prognosis in stage II/III RC. A total of 1031 cases treated with upfront resection or a neoadjuvant treatment modality were included in their study. Oncologic outcomes were not correlated with the timely initiation of treatment [15]. We notice that 229 (22.2%) patients received direct surgery, which may obscure a positive result. A recent study demonstrated that waiting time from diagnosis to primary treatment exceeding 2 months could not serve as an independent factor for OS and DFS in RC undergoing upfront operation [8]. Treatment modality may be a potential confounder. Several studies also defined 1 month as the threshold for treatment delay. Lee et al. found a significant increase in the risk of mortality in CRC with longer time intervals (> 1 month) between diagnosis and initiation of therapy. A timely treatment less than 1 month after diagnosis was recommended for CRC, regardless of tumor stage [21]. However, two studies with a similar design did not observe a significant relationship, which was consistent with the results of the present study in the Chinese cohort [8, 9]. Conversely, an Australian study demonstrated that CRC patients had lower survival when treated less than 1 month after diagnosis [22]. This phenomenon can be attributed to the tendency to prioritize early treatment for more complex cases. They attributed this to a preference for early treatment in more complicated cases. Clinical physicians may prioritize patients with more severe and emergency conditions, such as rectal bleeding or obstruction, for faster treatment. These patients may also have poor survival. This type of confounding can weaken or even reverse the estimates from the analysis, erroneously suggesting that longer treatment delay is protective [23]. Against this background, emerging radiotherapy strategies, including high‐dose‐rate endorectal brachytherapy for locally advanced rectal cancer and high linear energy transfer radiotherapy combined with immunotherapy in malignant tumors, further emphasize that the potential benefits of advanced local and multimodal treatments may be particularly sensitive to delays in treatment initiation, reinforcing the clinical importance of timely care delivery [24, 25].

Age is recognized as a significant potential factor contributing to clinical delays. Previous studies compared intervals between younger and older CRC patients; they indicated that younger individuals always experienced longer pre‐diagnostic intervals and shorter treatment delays when significant differences were reported [26, 27]. A tendency for longer diagnostic intervals in younger LARC patients was observed in our study. Younger adults are more likely to experience delays due to lower awareness of alarm symptoms, reluctance to seek treatment, as well as misdiagnosis and difficulties in accessing healthcare, associated with longer diagnostic delays and more advanced diseases [28, 29]. Interestingly, older age was found to be a poor prognostic factor for LARC. We consider that timely treatment may compensate for this disadvantage in the Chinese cohort, as almost all Chinese cases received therapy within one month. Additionally, a shorter treatment delay was also observed in younger LARC patients compared with older individuals in the American cohort, which is consistent with previous findings [30, 31]. Therefore, prompt treatment is important for these patients, regardless of their age.

Strengths of this study include the large sample size of LARC patients uniformly treated with neoadjuvant therapy, which helps minimize confounding related to disease stage and therapeutic modality. Both prediagnostic and treatment delay were examined, allowing for a comprehensive assessment of clinical delays. Moreover, by incorporating patients from both China and the United States, we were able to identify notable differences in treatment delay between the two countries and explore the relationship between age and clinical delay.

Several limitations should also be acknowledged. First, as a retrospective study, our analysis is inherently constrained by the completeness and accuracy of electronic health records and is susceptible to selection bias. Accordingly, we were unable to account for potentially important confounders such as education level, lifestyle factors, and socioeconomic status. The absence of these variables may introduce residual confounding and could partially influence the associations observed between diagnostic or treatment delays and survival. It is also possible that the ‘waiting time paradox’ would be attenuated if more detailed clinical covariates were captured, as patients with more aggressive disease or acute symptoms often receive expedited treatment yet inherently have poorer outcomes. Second, recall bias and limited awareness of symptom onset may influence the accuracy of reported diagnostic intervals. In addition, although we excluded cases with missing onset dates and applied standardized rules for handling approximate recall, the possibility of recall bias remains and may have contributed to measurement variability in diagnostic delay. Third, although the 5‐month diagnostic delay threshold was generated statistically using X‐tile, it also aligns with median diagnostic intervals reported in Western cohorts (4.4–4.6 months). Delays beyond approximately 4–6 months are often associated with more aggressive tumor biology or persistent barriers to accessing healthcare, supporting the clinical plausibility of this threshold. Fourth, because the SEER database lacks diagnostic delay information, the American cohort differs fundamentally from the Chinese dataset in terms of available variables. As a result, the two national cohorts address related but not fully comparable clinical questions, and this inherent heterogeneity weakens the strength of direct cross‐country comparisons. Therefore, caution is warranted when interpreting differences in clinical delay and outcomes at the national level.

## 5 Conclusion

In summary, this retrospective study found that a longer diagnostic delay from symptom onset to diagnosis and a prolonged treatment delay between diagnosis and treatment were associated with poorer survival in LARC. A less than 5‐month diagnostic interval and a 2‐month therapeutic interval may be safe for LARC patients. This study provides a reference for LARC patients who could not obtain a timely diagnosis and treatment. Prompt and accurate diagnosis, along with timely and effective therapy, are essential for improving the management of LARC.

## Author Contributions

Yutian Zhao: Cconceptualization, data curation, and writing – original draft; Jiahao Zhu: Fformal analysis, visualization, software, and writing – review and editing; Benjie Xu: Iinvestigation; Peipei Shen: Mmethodology; Fei Xu: Rresources and funding acquisition; Shengjun Ji: Vvalidation; Bo Yang: Pproject administration; and Leyuan Zhou: Vvalidation and funding acquisition. All authors have read and agreed to the published version of the manuscript.

## Funding

This work was supported by the Wuxi Taihu Lake Talent Plan, Leading Talents in Medical and Health Profession. Wuxi Municipal Health Commission Youth Project, Q202517.

## Ethics Statement

The present study adhered to the Declaration of Helsinki and was approved by the Affiliated Hospital of Jiangnan University, the Affiliated Suzhou Hospital of Nanjing Medical University, and the Harbin Medical University Cancer Hospital. Part of the data used in this study was obtained from the publicly available SEER database, which contains only de‐identified patient information. As such, ethical approval was not required.

## Consent

The patients/participants provided their written informed consent in three corresponding hospitals. Part of the data used in this study was obtained from the publicly available SEER database, which contains only de‐identified patient information. As such, informed consent was not required.

## Conflicts of Interest

The authors declare no conflicts of interest.

## Supporting information


**Table S1:** Baseline characteristics in Chinese patients with locally advanced rectal cancer.
**Figure S1:** Population distribution and survival difference between the short diagnostic delay group (indigo) and the long diagnostic delay group (gray) in Chinese patients with locally advanced rectal cancer when the threshold was set as 1 month. (Left) Population distribution (N = 45 vs. N = 405). (Middle) Overall survival (p = 0.655). (Right) Disease‐specific survival (p = 0.999).
**Figure S2:** Population distribution and survival difference between the short diagnostic delay group (indigo) and the long diagnostic delay group (gray) in Chinese patients with locally advanced rectal cancer when the threshold was set as 1 month. (Left) Population distribution (N = 97 vs. N = 353). (Middle) Overall survival (p = 0.752). (Right) Disease‐specific survival (p = 0.751).
**Figure S3:** Population distribution and survival difference between the short diagnostic delay group (indigo) and the long diagnostic delay group (gray) in Chinese patients with locally advanced rectal cancer when the threshold was set as 1 month. (Left) Population distribution (N = 173 vs. N = 277). (Middle) Overall survival (p = 0.073). (Right) Disease‐specific survival (p = 0.061).
**Figure S4:** Population distribution and survival difference between the short diagnostic delay group (indigo) and the long diagnostic delay group (gray) in Chinese patients with locally advanced rectal cancer when the threshold was set as 1 month. (Left) Population distribution (N = 222 vs. N = 228). (Middle) Overall survival (p = 0.065). (Right) Disease‐specific survival (p = 0.221).
**Figure S5:** Population distribution and survival difference between the short diagnostic delay group (indigo) and the long diagnostic delay group (gray) in Chinese patients with locally advanced rectal cancer when the threshold was set as 1 month. (Left) Population distribution (N = 289 vs. N = 161). (Middle) Overall survival (p = 0.029). (Right) Disease‐specific survival (p = 0.040).
**Figure S6:** Difference of treatment response between the short treatment delay group (STDG) and the middle treatment delay group (MTDG) in Chinese locally advanced rectal cancer patients.

## Data Availability

The data that support the findings of this study are available from the corresponding author upon reasonable request.
