# Supplementary material for: Influence of Diagnostic and Treatment Delays on Survival Outcomes in Patients With Locally Advanced Rectal Cancer: A Multi‐Center Retrospective Cohort Study
Source: Cancer Med. 2026 Mar 23;15(3):e71744. doi: 10.1002/cam4.71744 (PMC13140271; doi:10.1002/cam4.71744)
Supplement: Supplementary file 1 — Table S1: Baseline characteristics in Chinese patients with locally advanced rectal cancer. Figure S1: Population distribution and survival difference between the short diagnostic delay group (indigo) and the long diagnostic delay group (gray) in Chinese patients with locally advanced rectal cancer when the threshold was set as 1 month. (Left) Population distribution (N = 45 vs. N = 405). (Middle) Overall survival (p = 0.655). (Right) Disease‐specific survival (p = 0.999). Figure S2: Population distribution and survival difference between the short diagnostic delay group (indigo) and the long diagnostic delay group (gray) in Chinese patients with locally advanced rectal cancer when the threshold was set as 1 month. (Left) Population distribution (N = 97 vs. N = 353). (Middle) Overall survival (p = 0.752). (Right) Disease‐specific survival (p = 0.751). Figure S3: Population distribution and survival difference between the short diagnostic delay group (indigo) and the long diagnostic delay group (gray) in Chinese patients with locally advanced rectal cancer when the threshold was set as 1 month. (Left) Population distribution (N = 173 vs. N = 277). (Middle) Overall survival (p = 0.073). (Right) Disease‐specific survival (p = 0.061). Figure S4: Population distribution and survival difference between the short diagnostic delay group (indigo) and the long diagnostic delay group (gray) in Chinese patients with locally advanced rectal cancer when the threshold was set as 1 month. (Left) Population distribution (N = 222 vs. N = 228). (Middle) Overall survival (p = 0.065). (Right) Disease‐specific survival (p = 0.221). Figure S5: Population distribution and survival difference between the short diagnostic delay group (indigo) and the long diagnostic delay group (gray) in Chinese patients with locally advanced rectal cancer when the threshold was set as 1 month. (Left) Population distribution (N = 289 vs. N = 161). (Middle) Overall survival (p = 0.029). (Right) Disease‐specif [file CAM4-15-e71744-s001.docx]

**Influence of diagnostic and treatment delays on survival outcomes in patients with locally advanced rectal cancer: A multi-center retrospective cohort study**

Yutian Zhao, Jiahao Zhu, Benjie Xu, Peipei Shen, Fei Xu, Bo Yang, Shengjun Ji, Leyuan Zhou

**Supplementary Tables and Figures**

Table S1. Baseline characteristics in Chinese patients with locally advanced rectual cancer.

| Characteristics | Chinese cohort (n=450) | | |
| --- | --- | --- | --- |
|  | STDG (n=429) | MTDG (n=21) | *P* |
| Gender |  |  | 0.52 |
| Female | 152 (35.4%) | 6 (28.6%) |  |
| Male | 277 (64.6%) | 15 (71.4%) |  |
| Age (Years) |  |  | 0.107 |
| Median (IQR) | 58 (52 to 64) | 57 (51 to 59) |  |
| Year of diagnosis |  |  | 0.147 |
| 2010-2013 | 25 (5.8%) | 2 (9.5%) |  |
| 2014-2016 | 135 (31.5%) | 3 (14.3%) |  |
| 2017-2019 | 269 (62.7%) | 16 (76.2%) |  |
| Histology |  |  | 0.085 |
| Adenocarcinoma | 409 (95.3%) | 18 (85.7%) |  |
| Mucinous adenocarcinoma | 20 (4.7%) | 3 (14.3%) |  |
| Differentiation |  |  | 0.717 |
| Well | 43 (10.0%) | 3 (14.3%) |  |
| Moderate | 340 (79.3%) | 16 (76.2%) |  |
| Poor | 46 (10.7%) | 2 (9.5%) |  |
| Pretreatment CEA |  |  | 0.68 |
| Normal | 246 (57.3%) | 13 (61.9%) |  |
| Elevated | 183 (42.7%) | 8 (38.1%) |  |
| cT stage |  |  | 0.708 |
| cT2 | 19 (4.4%) | 0 (0.0%) |  |
| cT3 | 288 (67.1%) | 16 (76.2%) |  |
| cT4 | 122 (28.4%) | 5 (23.8%) |  |
| cN stage |  |  | 0.066 |
| cN0 | 135 (31.5%) | 12 (57.1%) |  |
| cN1 | 212 (49.4%) | 7 (33.3%) |  |
| cN2 | 82 (19.1%) | 2 (9.5%) |  |
| cTNM |  |  | 0.154 |
| IIA | 99 (23.1%) | 9 (42.9%) |  |
| IIB | 29 (6.8%) | 2 (9.5%) |  |
| IIC | 7 (1.6%) | 1 (4.8%) |  |
| IIIA | 19 (4.4%) | 0 (0.0%) |  |
| IIIB | 180 (42.0%) | 7 (33.3%) |  |
| IIIC | 95 (22.1%) | 2 (9.5%) |  |
| pPNI |  |  | 0.398 |
| Negative | 393 (91.6%) | 21 (100.0%) |  |
| Positive | 36 (8.4%) | 0 (0.0%) |  |
| pTDs |  |  | 0.435 |
| Negative | 331 (77.2%) | 18 (85.7%) |  |
| Positive | 98 (22.8%) | 3 (14.3%) |  |

Abbreviations: STDG: Short treatment delay group; MTDG: Middle treatment delay group; IQR: Interquartile range; CEA: Carcinoembryonic antigen; cT: Clinical tumor stage; cN: Clinical lymph node stage; cTNM: Clinical tumor node metastasis system stage, lymph node, and metastasis; TDs: Tumor deposits; PNI: Perineural invasion;

**Figure S1.** Population distribution and survival difference between short diagnostic delay group (indigo) and long diagnostic delay group (grey) in Chinese patients with locally advanced rectal cancer when threshold was set as 1 month. (Left) Population distribution (N = 45 vs. N = 405). (Middle) Overall survival (*P* = 0.655). (Right) Disease specific survival (*P* = 0.999).


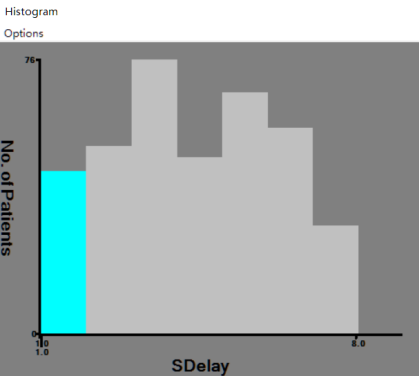

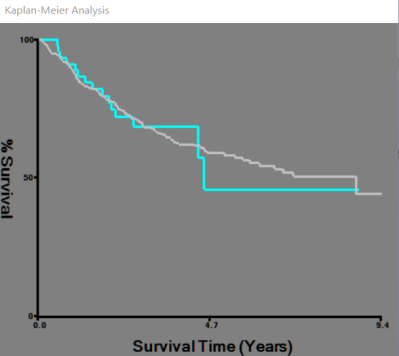

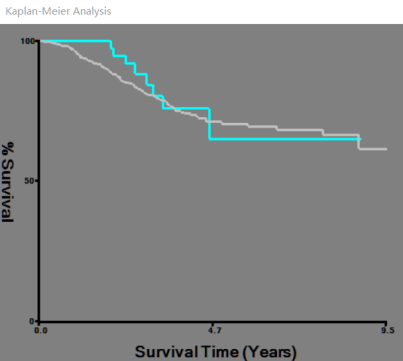


**Figure S2.** Population distribution and survival difference between short diagnostic delay group (indigo) and long diagnostic delay group (grey) in Chinese patients with locally advanced rectal cancer when threshold was set as 1 month. (Left) Population distribution (N = 97 vs. N = 353). (Middle) Overall survival (*P* = 0.752). (Right) Disease specific survival (*P* = 0.751).


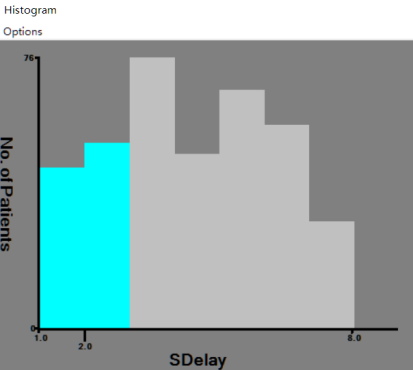

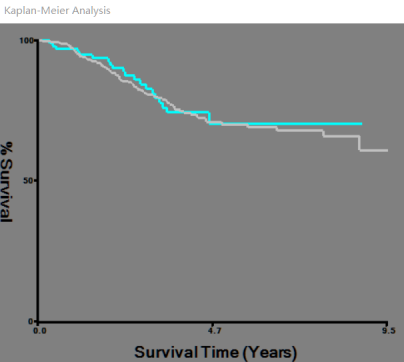

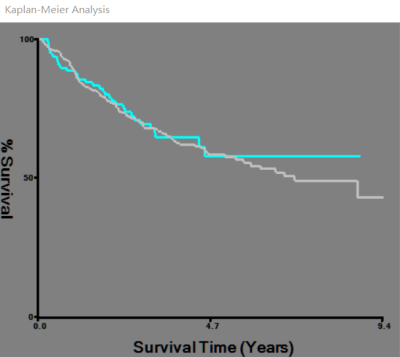


**Figure S3.** Population distribution and survival difference between short diagnostic delay group (indigo) and long diagnostic delay group (grey) in Chinese patients with locally advanced rectal cancer when threshold was set as 1 month. (Left) Population distribution (N = 173 vs. N = 277). (Middle) Overall survival (*P* = 0.073). (Right) Disease specific survival (*P* = 0.061).


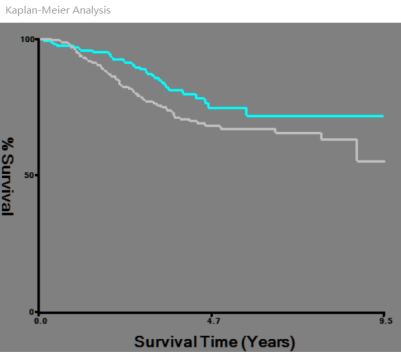

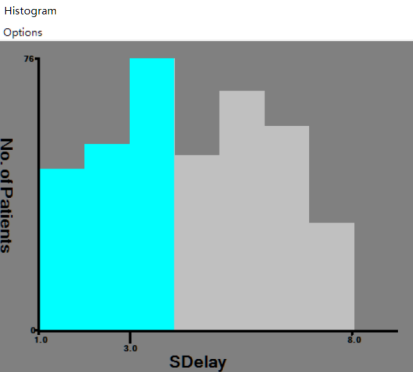

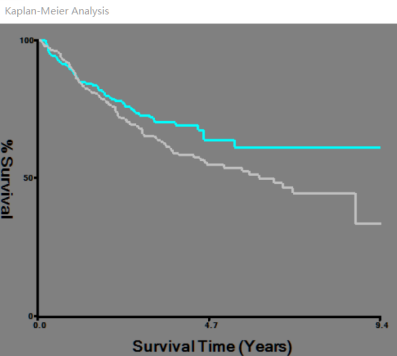


**Figure S4.** Population distribution and survival difference between short diagnostic delay group (indigo) and long diagnostic delay group (grey) in Chinese patients with locally advanced rectal cancer when threshold was set as 1 month. (Left) Population distribution (N = 222 vs. N = 228). (Middle) Overall survival (*P* = 0.065). (Right) Disease specific survival (*P* = 0.221).


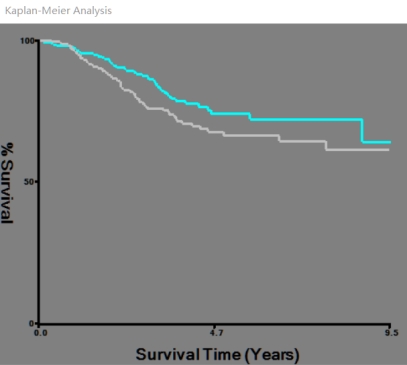

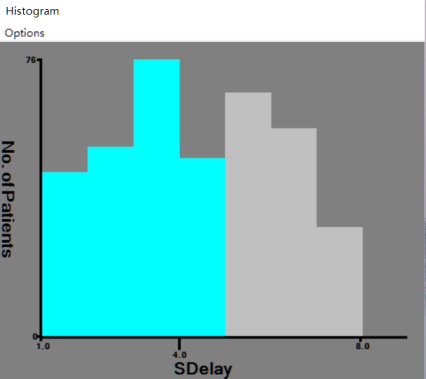

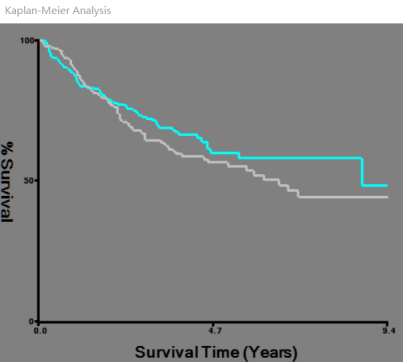


**Figure S5.** Population distribution and survival difference between short diagnostic delay group (indigo) and long diagnostic delay group (grey) in Chinese patients with locally advanced rectal cancer when threshold was set as 1 month. (Left) Population distribution (N = 289 vs. N = 161). (Middle) Overall survival (*P* = 0.029). (Right) Disease specific survival (*P* = 0.040).


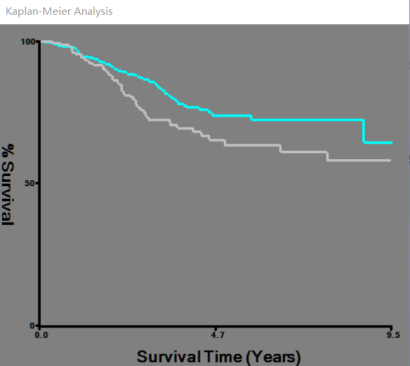

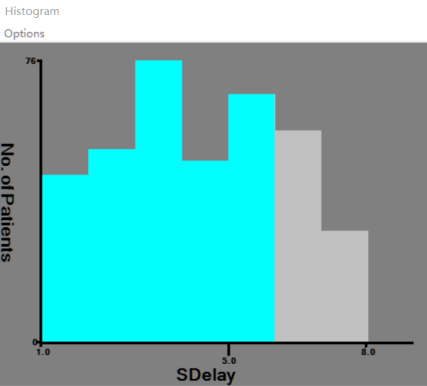

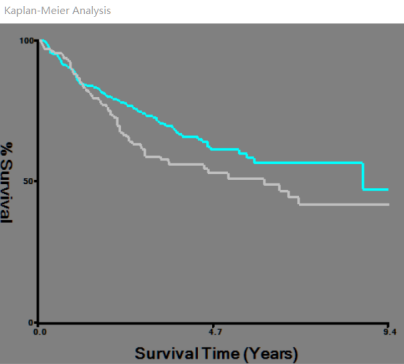


**
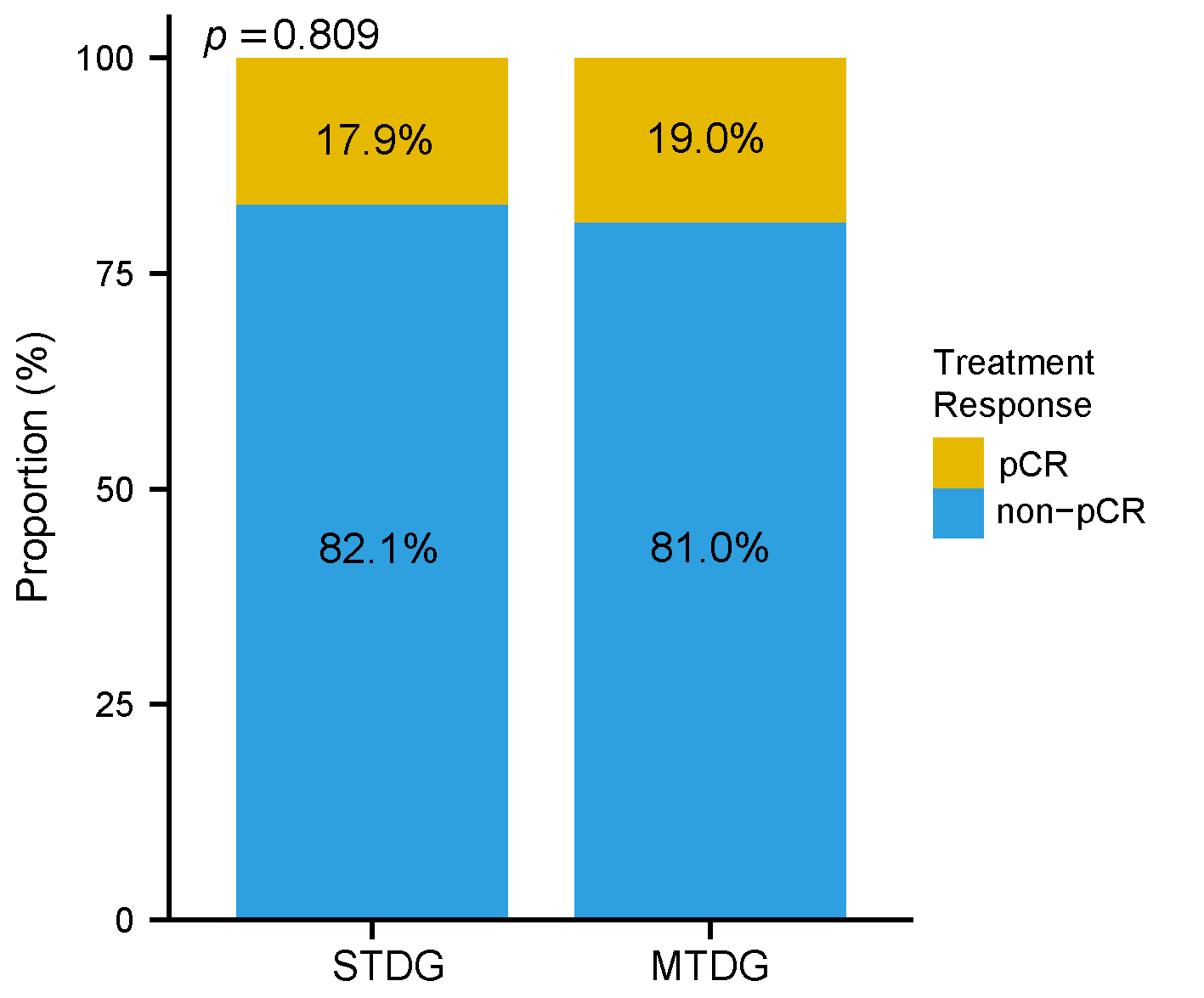
Figure S6.** Difference of treatment response between short treatment delay group (STDG) and middle treatment delay group (MTDG) in Chinese locally advanced rectal cancer patients.
